# Markers of T-cell dysfunction and not inflammaging predict the waning of humoral responses to SARS-CoV-2 mRNA booster vaccination in people with HIV

**DOI:** 10.1097/QAD.0000000000004010

**Published:** 2024-10-31

**Authors:** Matteo Augello, Valeria Bono, Roberta Rovito, Andrea Santoro, Camilla Tincati, Giulia Marchetti

**Affiliations:** Clinic of Infectious Diseases and Tropical Medicine, San Paolo Hospital, ASST Santi Paolo e Carlo, Department of Health Sciences, University of Milan, Milan, Italy.

## Abstract

In this prospective longitudinal study, we evaluated the durability of humoral responses to SARS-CoV-2 mRNA booster vaccination in 93 people with HIV, exploring the possible role of T-cell dysfunction and inflammaging biomarkers in predicting antibody waning. We found that, despite a negligible influence of the inflammaging *milieu*, low CD4/CD8 ratio and CD4^+^CD127^+^ percentage as well as high CD8^+^CD38^+^CD45RO^+^ percentage are associated with faster antibody waning, in turn contributing to our understanding of the determinants of COVID-19 vaccine-elicited immune response in this population.

People with HIV (PWH) may suffer worse COVID-19 outcomes compared to the general population [1,2]. Preventing SARS-CoV-2 infection remains a relevant goal in PWH, also in the endemic phase of COVID-19, since it has been associated with a higher incidence of long COVID-19 and subsequent major cardiovascular events in this population independently of its severity [3,4]. Indeed, the widespread use of SARS-CoV-2 vaccination considerably decreased the risk of infection and severe disease by eliciting strong humoral and T-cell immunity [1,5]. However, while vaccine-induced T-cell responses are durable, thus ensuring long-lasting protection from severe disease, humoral immunity wanes over time, in turn affecting the protection against SARS-CoV-2 infection [6,7]. Furthermore, while factors influencing SARS-CoV-2 vaccines immunogenicity have been extensively characterized in PWH [1,5,8–10], determinants of antibody waning are largely unknown.

Persistent T-cell dysregulation and inflammaging despite virologically-effective antiretroviral therapy (ART) are hallmarks of HIV infection, and have been associated with adverse immunological and clinical outcomes [11], as well as poor vaccine immunogenicity [12–15].

We therefore aimed to assess the durability of humoral responses to a SARS-CoV-2 mRNA booster in PWH and to explore the possible role of baseline HIV-related T-cell dysfunction and inflammaging *milieu* in predicting antibody waning.

In this prospective longitudinal study conducted at the Clinic of Infectious Diseases and Tropical Medicine at San Paolo Hospital in Milan, Italy, PWH on virologically-effective ART who received a monovalent mRNA booster (Moderna Spikevax) 6 months after the primary cycle were consecutively enrolled, and followed-up from baseline (T0) to 1 month (T1) and 6 months (T2) after the booster administration.

Anti-spike (S) immunoglobulin G (IgG) antibodies were quantitatively measured on serum samples at each time point by the DiaSorin LIAISON SARS-CoV-2 TrimericS IgG assay. Serum anti-nucleocapsid (N) IgG were semi-quantitatively determined by the EUROIMMUN Anti-SARS-CoV-2 NCP ELISA IgG to track SARS-CoV-2 infection at baseline and throughout the follow-up period.

To assess T-cell dysfunction, CD4/CD8 ratio, as well as percentages of CD4^+^CD127^+^ T-cells (functionally-competent CD4^+^ T-cells) and CD8^+^CD38^+^CD45RO^+^ T-cells (primed activated CD8^+^ T-cells) were measured on blood samples at baseline by flow cytometry (Figure S1, Supplemental Digital Content). Markers of inflammaging were quantified in plasma at T0 by Luminex assay [tumor necrosis factor alpha (TNF-α), IP-10, interleukin 2 (IL-2), IL-4, IL-17A, IL-6, IL-8, sCD14, sCD163, GDF-15, MMP-9, TIMP-1] or ELISA (thymosin-α1, Elabscience Human Thymosin-α1 ELISA kit). A composite “inflammaging score” ranging from 0 to 13 was calculated as the number of biomarkers with an abnormal level (at or above the 75th percentile for TNF-α, IP-10, IL-17A, IL-6, IL-8, sCD14, sCD163, GDF-15, MMP-9, and TIMP-1; at or below the 25th percentile for IL-2, IL-4, and thymosin-α1), similar as previously described [16].

Demographic and clinical characteristics of the study population were also recorded at baseline.

Repeated measures ANOVA, Pearson correlation test, and multivariable linear regression were performed using GraphPad Prism v10.

Ninety-three PWH were recruited and followed-up between September 2021 and July 2022. Median age was 53 [interquartile range (IQR): 46–59] years, and 76 (81.7%) were males. Median CD4^+^ T-cell *nadir* was 226 (IQR: 53–353) cells/μl; current CD4^+^ T-cell count was 728 (IQR: 511–920) cells/μl, with a median CD4/CD8 ratio of 0.76 (IQR: 0.60–1.06). All participants have been on ART for a median of 114 (IQR: 67–186) months, and were virologically-suppressed (HIV viremia < 20 copies/ml). Study participants characteristics are detailed in Table S1, Supplemental Digital Content.

Four PWH had positive anti-N IgG at T0, four developed anti-N IgG positivity between T0 and T1, and four more between T1 and T2.

Anti-S IgG antibodies were significantly increased 1 month after the booster [median: 3.99 (IQR: 3.71–4.27) vs. 2.94 (2.62–3.17) log_10_(BAU/ml), *P* < 0.0001], with a subsequent 6-month decay at levels that were still above baseline [3.36 (3.15–3.78) log_10_(BAU/ml), *P* < 0.0001] (Fig. 1a).

**Fig. 1:**
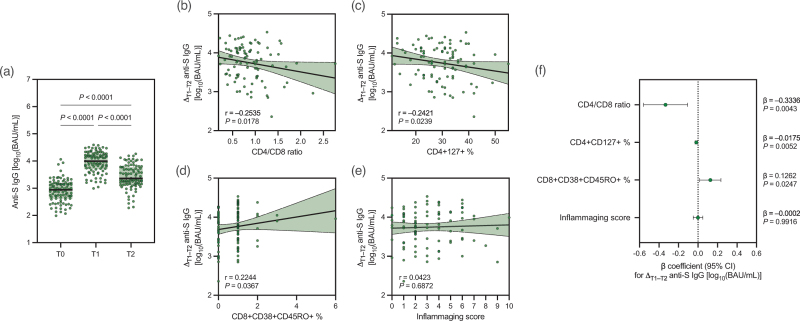
Anti-S IgG antibodies concentrations and associations with markers of T-cell dysfunction and inflammaging.

(a) Trajectory of anti-S IgG antibodies concentration following the booster. T0: baseline (day of booster administration); T1: 1 month after the booster; T2: 6 months after the booster; *circles*: individual values; *black bars*: median values; *boxes*: interquartile ranges; *statistical analysis*: repeated measures ANOVA. (b–e) Correlations between waning of anti-S IgG antibodies, measured as the difference between T1 and T2 levels (Δ_T1–T2_), and CD4/CD8 ratio, CD4^+^CD127^+^ and CD8^+^CD38^+^CD45RO^+^ percentages, or inflammaging score. *Circles*: individual values; *solid black line*: simple linear regression line; *shadowed error bands within dashed lines*: interquartile ranges; *statistical analysis*: Pearson correlation test. (f) Forest plot showing associations between waning of anti-S IgG antibodies (Δ_T1–T2_) and CD4/CD8 ratio, CD4^+^CD127^+^ percentage, CD8^+^CD38^+^CD45RO^+^ percentage, or inflammaging score. *Circles*: *β* coefficients; *error bars*: 95% confidence intervals (95% CI); *statistical analysis*: multivariable linear regression (adjusted for age, sex, Charlson Comorbidity Index, obesity, smoke, anti-N IgG status at baseline and during follow-up).

Anti-S IgG waning, measured as the difference between T1 and T2 levels (Δ_T1–T2_), was negatively correlated with CD4/CD8 ratio (*r* = –0.2535, *P* = 0.0178) and CD4^+^CD127^+^ percentage (*r* = –0.2421, *P* = 0.0239), while positively with CD8^+^CD38^+^CD45RO^+^ percentage (*r* = 0.2244, *P* = 0.0367) (Fig. 1b–d). Noteworthy, when controlling for potential confounders/predictors (i.e., age, sex, Charlson Comorbidity Index, obesity, smoke, anti-N IgG positivity at baseline and/or during follow-up), anti-S IgG waning was confirmed negatively associated with CD4/CD8 ratio [*β* = –0.3336 (95% CI: –0.5594, –0.1079), *P* = 0.0043] and CD4^+^CD127^+^ percentage [*β* = –0.0175 (95% CI: –0.0297, –0.0054), *P* = 0.0052], while positively with CD8^+^CD38^+^CD45RO^+^ percentage [*β* = 0.1262 (95% CI: 0.0165, 0.2360), *P* = 0.0247] (Fig. 1f). Similar results were obtained when performing a sensitivity analysis after excluding PWH with positive anti-N IgG antibodies at any time point (Table S2, Supplemental Digital Content).

Low CD4/CD8 ratio and CD4^+^CD127^+^ T-cell percentage as well as high CD8^+^CD38^+^CD45RO^+^ percentage have been associated with unfavorable immunological and clinical outcomes in HIV infection [17–19]. Additionally, CD4/CD8 ratio has been reported to negatively correlate with SARS-CoV-2 vaccines immunogenicity in PWH [5,8,20]. Our findings argue that such peculiar HIV-related anomalies are predictive of faster antibody waning following SARS-CoV-2 booster vaccination, thus potentially expanding the utility of these markers in this setting.

By contrast, when assessing inflammaging score, we found that it was not associated with anti-S IgG waning, either at univariable (*r* = 0.0423, *P* = 0.6872) (Fig. 1e) or multivariable analysis [*β* = –0.0002 (95% CI: –0.0492, 0.0487), *P* = 0.9916] (Fig. 1f). A lack of association between anti-S IgG waning and inflammaging was also found when analyzing inflammaging markers individually (Figure S2, Supplemental Digital Content).

This observation was somehow unexpected, given previous reports showing an association of reduced vaccine-elicited serological memory with markers of inflammation [21,22] and thymic dysfunction [23]. However, these studies assessed immune responses to different vaccines or in different populations/models, and thus cannot be directly translated to our setting.

Some limitations need to be acknowledged in this study. Firstly, the sample size was relatively small, thus potentially hindering the generalizability of such findings. Furthermore, the follow-up was limited to 6 months, hence longer periods should be included in future studies to ascertain the actual long-term durability of vaccine-induced humoral immunity and thus the optimal timing for boosters administration. Besides, while the present study was specifically designed to explore whether markers of T-cell dysfunction and inflammaging measured at baseline are able to predict waning of vaccine-elicited humoral responses later on, it would be interesting for future studies to evaluate whether administration of vaccine boosters may have an impact on T-cell phenotypes and cytokine *milieu* over time.

In summary, our study points to low CD4/CD8 ratio and CD4^+^CD127^+^ T-cells as well as high CD8^+^CD38^+^CD45RO^+^ T-cells as factors negatively affecting the durability of humoral responses to SARS-CoV-2 mRNA booster in PWH, in all suggesting the potential for T-cell dysfunction biomarkers in the assessment of individuals to be prioritized for future SARS-CoV-2 boosters.

## Acknowledgements

We are grateful to all the individuals enrolled in this study who agreed to participate to this research. Our special thanks also go to all the physicians and nurses at the Clinic of Infectious Diseases and Tropical Medicine at San Paolo Hospital in Milan who helped in patients’ care and enrollment. We are also thankful to Alessandro Cozzi-Lepri for his precious suggestions for statistical analyses design.

Authors contributions: M.A. conceived and designed the study, collected clinical and laboratory data, analyzed and interpreted the data, designed the figures, and wrote the manuscript. V.B., R.R., and A.S. contributed to data collection. C.T. contributed to the critical revision of the manuscript. G.M. conceived and supervised the study, interpreted the data, and wrote the manuscript.

Funding: This study was supported by funding from the European Union‘s Horizon 2020 Research and Innovation Program under grant agreement no. 101016167 within the ORCHESTRA project (Connecting European Cohorts to Increase Common and Effective Response to SARS-CoV-2 Pandemic), and grant agreement no. 101046016 within the EuCARE project (European Cohorts of Patients and Schools to Advance Response to Epidemics).

### Conflicts of interest

Authors have no conflicts of interest related to this work to disclose.

## Supplementary Material

Supplemental Digital Content
