# Supplementary material for: Markers of T-cell dysfunction and not inflammaging predict the waning of humoral responses to SARS-CoV-2 mRNA booster vaccination in people with HIV
Source: AIDS. 2024 Oct 31;38(14):1987–90. doi: 10.1097/QAD.0000000000004010 (PMC11556865; doi:10.1097/QAD.0000000000004010)

Supplementary material

Table S1. Demographic, clinical, and HIV-related characteristics of study participants at baseline.

**Characteristics**	**PWH (*n*=93)**
**Age, years**, median (IQR)	53 (46–59)
**Sex at birth**, *n* (%)	
Male	76 (81.7)
Female	17 (18.3)
**Ethnicity**, *n* (%)	
Caucasian	81 (87.1)
African	5 (5.4)
Latin	5 (5.4)
Asian	2 (2.1)
**Epidemiology**, *n* (%)	
MSM	47 (50.5)
MSW/WSM	30 (32.3)
IDU	16 (17.2)
**Comorbidities**, *n* (%)	
Cardiovascular diseases	26 (28)
Chronic kidney diseases	13 (14)
COPD/Asthma	10 (10.8)
Hepatic diseases	11 (11.8)
Peptic ulcer	9 (9.7)
Diabetes	16 (17.2)
Neurologic diseases	12 (12.9)
Previous leukemia/lymphoma	12 (12.9)
Previous solid cancer	12 (12.9)
Autoimmune diseases	14 (15.1)
**Obesity (BMI ≥30)**, *n* (%)	13 (14)
**Charlson Comorbidity Index**, median (IQR)	2 (1–3)
**Smoke**, *n* (%)	30 (32.3)
**Viro-immunologic parameters**, median (IQR)	
CD4 T-cell *nadir*, cells/μL	226 (53–353)
CD4 T-cell count, cells/μL	728 (511–920)
CD4 T-cell percentage	32 (25–38)
CD8 T-cell count, cells/μL	896 (661–1195)
CD8 T-cell percentage	41 (33–45)
CD4/CD8 ratio	0.76 (0.60–1.06)
**Previous AIDS diagnosis**, *n* (%)	60 (75)
**Undetectable HIV-RNA (<50 copies/mL)**, *n* (%)	93 (100)
**Time from HIV diagnosis, months**, median (IQR)	135 (83–309)
**Current ART regimen**, *n* (%)	
INSTI-based (triple)	44 (47.3)
INSTI-based (dual)	19 (20.4)
NNRTI-based	26 (28)
PI-based	4 (4.3)
**Duration of ART, months**, median (IQR)	114 (67–186)

Legend: *PWH*: people with HIV; *IQR*: interquartile range; *MSM*: men who have sex with men; *MSW*: men who have sex with women; *WSM*: women who have sex with men; *IDU*: injective drug use; *COPD*: chronic obstructive pulmonary disease; *BMI*: body mass index; *AIDS*: acquired immune deficiency syndrome; *ART*: antiretroviral therapy; *INSTI*: integrase strand-transfer inhibitor; *NNRTI*: non-nucleoside reverse transcriptase inhibitor.

Table S2. Sensitivity analysis after excluding PWH with positive anti-N IgG at any time point.

**Multivariable linear regression estimating β coefficients for the Δ_{T1-T2} anti-S IgG adjusted for age, sex, obesity, smoke, and Charlson Comorbidity Index**			
**Variable**	**β coefficient**	**95% CI**	***P* value**
CD4/CD8 ratio	–0.3349	–0.5705, –0.0992	0.0060
CD4+CD127+ %	–0.0170	–0.0299, –0.0040	0.0112
CD8+CD38+CD45RO+ %	0.1229	0.0029, 0.240	0.0448
Inflammaging score	0.0047	–0.0502, 0.0596	0.8641

Figure S1. CD4+, CD8+, CD4+CD127+, and CD8+CD38+CD45RO+ lymphocytes were quantified by flow cytometry. Briefly, flow cytometry samples were prepared with the automated BD FACSDuet™ Sample Preparation System by mixing 50 μL of fresh whole blood and 40 μL of fluorescently-labeled antibodies cocktail (CD45–V500, CD4–APC-Cy7, CD8–PerCP-Cy5.5, CD127–PE, CD38–APC, CD45RO–FITC) in BD Trucount™ Tubes. After a 20-minute incubation at room temperature, red blood cells were lysed with BD FACS™ Lysing Solution. Samples were then acquired on a BD FACSLyric™ Flow Cytometry System, and fcs files analysed with BD FACSuite™ Clinical Application software. The gating strategy for a representative sample (generated with FlowJo v10) is shown below.

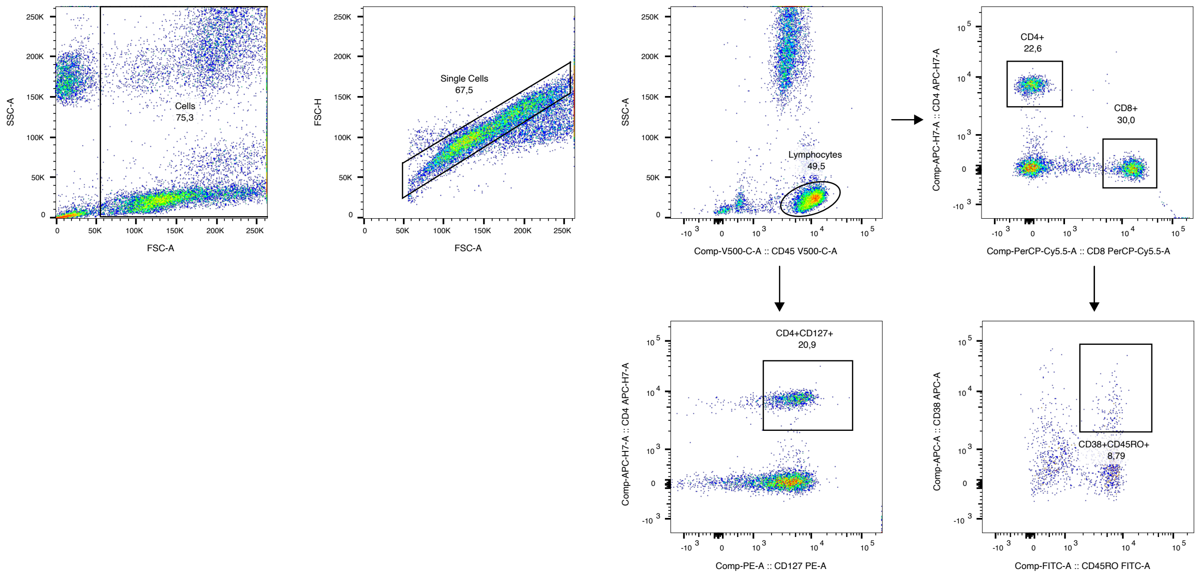

Figure S2. A. Correlations between waning of anti-S IgG antibodies, measured as the difference between T1 and T2 levels (Δ_{T1-T2}), and plasma concentration of inflammaging markers at baseline (T0). *Symbols*: individual values; *statistical analysis*: Pearson correlation test. **B.** Forest plot showing associations between waning of anti-S IgG antibodies (Δ_{T1-T2}) and inflammaging markers. *Circles*: β coefficients; *error bars*: 95% confidence intervals (95% CI); *statistical analysis*: multivariable linear regression (adjusted for age, sex, Charlson Comorbidity Index, obesity, smoke, anti-N IgG status at baseline and during follow-up).

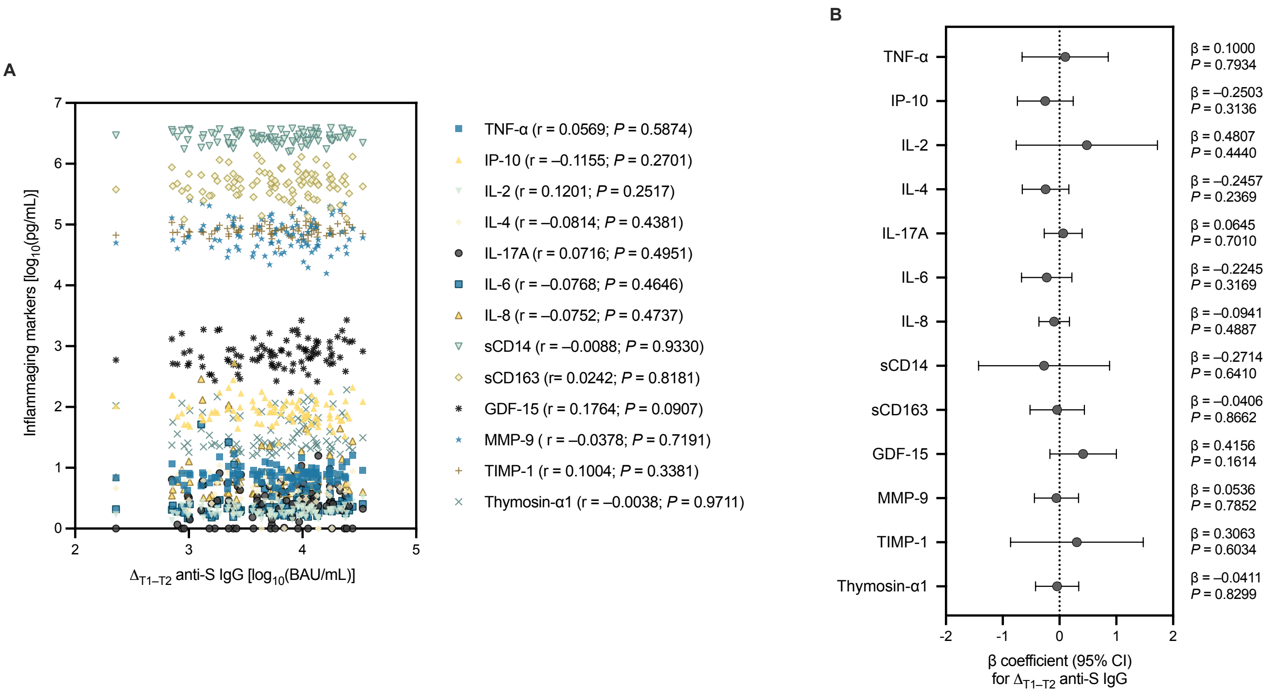

Supplement: Supplemental Digital Content [file aids-38-1987-s001.pdf]
